# Possible Association of Mutations in the *MEFV* Gene with the Intestinal Phenotype of Behçet’s Disease and Refractoriness to Treatment

**DOI:** 10.3390/jcm12093131

**Published:** 2023-04-26

**Authors:** Yoki Furuta, Ryosuke Gushima, Hideaki Naoe, Munenori Honda, Yuiko Tsuruta, Katsuya Nagaoka, Takehisa Watanabe, Masakuni Tateyama, Nahoko Fujimoto, Shinya Hirata, Eiko Miyagawa, Komei Sakata, Yumiko Mizuhashi, Mikako Iwakura, Masayuki Murai, Masao Matsuoka, Yoshihiro Komohara, Yasuhito Tanaka

**Affiliations:** 1 Department of Gastroenterology and Hepatology, Graduate School of Medical Sciences, Kumamoto University, Kumamoto 860-8556, Japan; 2 Department of Hematology, Rheumatology and Infectious Diseases, School of Medicine, Kumamoto University, Kumamoto 860-8556, Japan; 3 Department of Cell Pathology, Graduate School of Medical Sciences, Faculty of Life Sciences, Kumamoto University, Kumamoto 860-8556, Japan

**Keywords:** inflammatory bowel disease, intestinal Behçet’s disease, interleukin-1β, Mediterranean fever gene

## Abstract

Background: Mediterranean fever (*MEFV*) gene mutations are responsible for familial Mediterranean fever (FMF) and associated with other inflammatory diseases. However, the effects of *MEFV* gene mutations on intestinal Behçet’s disease (BD) are unknown. In this study, we investigated these mutations and clinical features in patients with intestinal BD. Methods: *MEFV* gene analysis was performed in 16 patients with intestinal BD, 10 with BD without intestinal lesions, and 50 healthy controls. Clinical features of patients with intestinal BD were retrospectively assessed. Results: The rates of *MEFV* gene mutations in patients with intestinal BD, BD without intestinal lesions, and healthy controls were 75%, 50%, and 38%, respectively. Only 2 of 12 patients with intestinal BD harboring *MEFV* gene mutations (17%) were controlled without immunosuppressive treatment, while 8 patients (67%) required therapy with tumor necrosis factor (TNF) inhibitors. Among patients with intestinal BD without *MEFV* gene mutations (four patients), three (75%) were controlled by the administration of 5-aminosalicylic acid with or without colchicine, and one (25%) required TNF inhibitors. All patients who underwent intestinal resection had *MEFV* gene mutations. Immunohistochemical analysis and in situ hybridization with interleukin-1β (IL-1β) showed a high expression of IL-1β only in injured areas, suggesting that IL-1β may be involved in the formation of ulcers in patients with intestinal BD carrying *MEFV* gene mutations. Conclusion: Mutations in the *MEFV* gene may be associated with intestinal lesions of BD and refractoriness to treatment.

## 1. Introduction

Behçet’s disease (BD) is a rare chronic inflammatory disorder with unknown etiology; it is characterized by four major manifestations, namely recurrent oral ulcers, genital ulcers, uveitis, and skin lesions [1]. BD also affects several organs, including the vessels, central nervous, and gastrointestinal systems. The intestinal manifestation of BD reduces the quality of life due to abdominal pain and hemorrhage, and it occasionally causes intestinal perforation, which can be a life-threatening condition [2]. The incidence of intestinal manifestation in BD ranges 3–16%, and it is relatively higher in countries of East Asia, such as Japan and Korea; conversely, intestinal involvement in BD is rare in Mediterranean countries [3,4,5,6]. Owing to racial and geographical differences in the incidence of intestinal manifestation, genetic factors may be associated with the development of intestinal lesions. However, only a few reports have partly analyzed genetic risk factors for intestinal involvement of BD [4,7].

Familial Mediterranean fever (FMF), which is an autosomal autoinflammatory disease, shares some clinical features with BD. Both diseases are prevalent in the Mediterranean region, respond well to treatment with colchicine, and may coexist [8,9]. In addition, the gene which is responsible for FMF—Mediterranean fever (*MEFV*) gene—has been identified as a susceptibility gene for BD [10]. The *MEFV* gene encodes pyrin, which is part of a protein complex termed inflammasome and negatively regulates its activation [11]. Activation of the inflammasome causes the cleavage of caspase-1 (CASP1), leading to the conversion of pro interleukin-1β (IL-1β) into its active form (i.e., IL-1β). Therefore, *MEFV* gene mutations in exon 1–3, 5, and 10 cause dysfunction of pyrin and result in excessive inflammation [12]. Colchicine is a tricyclic alkaloid which binds tubulin, thereby resulting in the regulation of IL-1β expression in neutrophils. Therefore, colchicine is a first-line drug for the treatment of patients with FMF. According to recent reports, *MEFV* gene mutations may act as disease-modifying factors in neuro-BD [13]. Moreover, several studies showed that *MEFV* gene mutations were associated with intestinal inflammation, and a new disease concept termed *MEFV* gene-related enterocolitis was recently proposed [14,15,16]. These *MEFV* gene-related enterocolitis has attracted considerable attention due to its good response to treatment with colchicine. Based on these findings, we hypothesized that *MEFV* gene mutations may be associated with the development of intestinal lesions in BD. In this study, we analyzed *MEFV* gene mutations in patients with BD and assessed their association with the development of intestinal lesions as well as their effects on the treatment of intestinal BD.

## 2. Materials and Methods

### 2.1. Subjects

This retrospective study was conducted at Kumamoto University Hospital. The study protocol was approved by the Institutional Review Board of Kumamoto University Hospital on 1 September 2020 (approval number: 453). This investigation was performed in accordance with the tenets of the Declaration of Helsinki. This study included patients diagnosed with BD who visited Kumamoto University Hospital between April 2018 and December 2020. In addition, healthy volunteers of Japanese origin were analyzed for MEFV gene mutations. The diagnosis of BD and clinical subtypes were determined according to the relevant Japanese diagnostic criteria [1]. The diagnosis of intestinal BD was reached based on the 2nd edition of consensus statements for the diagnosis of intestinal BD [17]. Patients with only intestinal manifestation and those who did not meet any major criteria of BD (i.e., simple ulcer) were excluded from this study. We also excluded patients with myelodysplastic syndrome harboring trisomy 8, which is considered a risk factor for a BD-like syndrome with intestinal lesions [18].

### 2.2. MEFV Gene Analysis

Genomic DNA was extracted from peripheral blood using a Puregene Blood Core Kit (Qiagen, Hilden, Germany). Analysis of the *MEFV* gene was conducted in all patients. Mutations in the five hotspot regions (i.e., exons 1, 2, 3, 5, and 10) were assessed according to the diagnostic criteria for FMF through polymerase chain reaction (PCR). Precise SNP numbers or location numbers were as follows: E84K (16: 3246339) in exon 1, L110P (rs11466018), E148Q (rs3743930), R202Q (16: 3244725) and G304R (16: 3244159) in exon 2, P369S (rs11466023) and R408Q (rs11466024) in exon 3, S503C (16: 3237096) in exon 5, M680I (rs28940580), M694V (rs61752717), M694V (rs61752717) and V726A (rs28940579) in exon 10. Amplified PCR products were analyzed by direct sequencing (DNA Analyzer 3130; Applied Biosystems, Foster City, CA, USA). The primers used for the PCR and sequence analysis were as follows: exon 1F: 5′-AGT TCA AGT TCA AGC TGC AG-3′; exon 1R: 5′-TGG ATT TTG GTA GAC CTG AG-3′; exon 2F: 5′-ATA TCC AAG GGG ATT CTC TC-3′; exon 2R: 5′-TAC ATT CAC CAG GCT GGT-3′; exon 3F: 5′-ACG GTA CCT GTG TGC GTG AT-3′; exon 3R: 5′-TCT GAG CCT CTG GAT TCA GC-3′; exon 5F: 5′-AAT CTA GGC CTT GAA GAG GC-3′; exon 5R: 5′-AGA CCT CAT CAA AGG GGT CT-3′; exon 10F: 5′-GAG GTG GAG TTG GAG ACA A-3′; and exon 10R: 5′-AGA GCA GCT GGC GAA TGT AT-3′. Annealing temperatures were as follows: exon 1: 58 °C; exon 2: 60 °C; exon 3: 62 °C; exon 5: 60 °C; and exon 10: 60 °C.

### 2.3. Cytokine Expression Analysis

For immunohistochemistry (IHC), sectioned formalin-fixed paraffin-embedded biopsy samples were deparaffinized and rehydrated, which was followed by heat-induced and enzymatic antigen retrieval. Subsequently, sections were blocked and incubated at 4 °C overnight with the following primary antibodies: anti-IL-1β antibody (1:100, 12242; Cell Signaling Technology, Inc., Danvers, MA, USA) or anti-IL-18 antibody (1:1000, ab243091; Abcam, Cambridge, UK). Thereafter, biotinated or horseradish peroxidase-conjugated secondary antibodies (HAF007; R&D Systems, Inc., Minneapolis, MN, USA) were used for detection. Anti-CD66b antibodies (1:100, 3929023, Biolegend, San Diego, CA, USA) were used for the detection of neutrophils. Images were captured using Axio Scope AI (ZEISS, Oberkochen, Germany).

For RNA in situ hybridization, the RNAScope Kit (Advanced Cell Diagnostics, Newark, CA, USA) was used to measure the mRNA expression in paraffin-embedded sections.

### 2.4. Statistical Analysis

Continuous data were compared using the Mann–Whitney *U*-test. Pearson’s χ^2^ test or Fisher’s exact test were used to analyze the categorical data and compare proportions. *p*-values < 0.05 denoted statistically significant differences. All analyses were performed using the SPSS software version 28.0 (IBM Corp., Armonk, NY, USA).

## 3. Results

### 3.1. Clinical Characteristics of Patients with BD

Twenty-six patients with BD were enrolled in this study, and Table 1 summarizes the characteristics of patients. For the 26 patients (19 males, 73%; seven females, 27%), the mean age at diagnosis was 34 years and the median duration of BD was 8.5 years. In the subtype classification, 5 (19%), 16 (62%), and 5 (19%) patients were diagnosed as the complete type, incomplete type, and suspected of BD, respectively. Regarding major symptoms, oral ulcers were observed in all patients, skin lesions were observed in 17 patients (65%), eye lesions were observed in 13 patients (50%), and genital ulcers were observed in 10 patients (38%). Gastrointestinal involvement was noted in 16 patients (62%). Of the 26 patients, 12 (46%) and 8 (31%) were positive for human leukocyte antigen (HLA)-B51 and HLA-A26, respectively. Two patients (8%) were positive for both HLA-B51 and HLA-A26.

### 3.2. Detection of MEFV Gene Mutations and Clinical Manifestations

**Figure 1:**
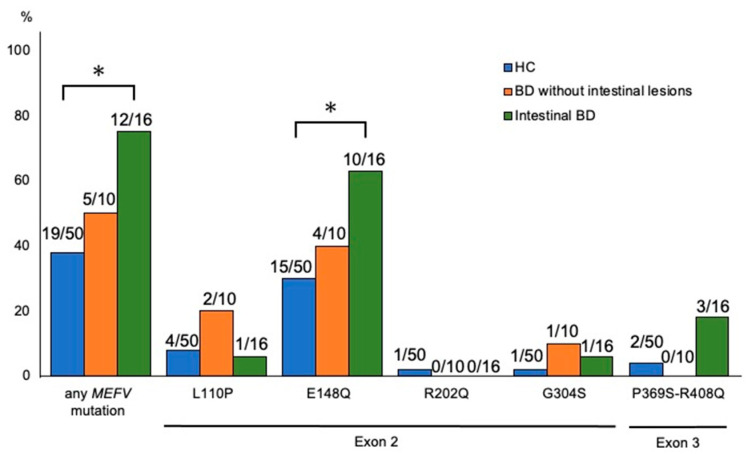
*MEFV* gene mutations in healthy controls (HC), patients with Behçet’s disease (BD) without intestinal lesions, and patients with intestinal BD. The incidence of mutations in the *MEFV* gene was compared between patients with intestinal BD (*n* = 16), BD patients without intestinal le-sions (*n* = 10), and HC (*n* = 50). * *p* < 0.05. *MEFV*, Mediterranean fever.

*MEFV* gene mutations were identified in 17 of the 26 patients with BD (65%) and the most common mutation was E148Q in exon 2 (14 patients, 54%) (Figure 1). Other mutations were L110P and G304S in exon 2 and P369S-R408Q in exon 3. Seven patients showed a homozygous mutation. Three were patients with intestinal BD patients and four were BD without intestinal lesions. Compound heterozygous mutation was identified in two patients with intestinal BD (Table 2).

Hence, *MEFV* gene mutations among the 16 patients with intestinal BD were identified in 12 patients with BD (75%), which was higher than those of 5 patients (50%) in 10 BD patients without intestinal lesions. *MEFV* gene mutations vary by region and country; thus, we analyzed Japanese origin 50 healthy controls for these mutations for comparison [11,19]. Compared with healthy controls (Japanese general population), the incidence of these gene mutations was significantly higher in patients with intestinal BD (75% vs. 38%, see Figure 1).

To assess the clinical impact of these gene mutations on BD with intestinal lesions, we classified the patients into two groups: *MEFV* mutation-positive (*MEFV*+) and *MEFV* mutation-negative (*MEFV*−) (Table 3). Patients in the *MEFV*− group were associated with a higher incidence of skin lesions than those in the *MEFV*+ group (100% vs. 33%, respectively, *p* = 0.077). We assessed the endoscopic findings of intestinal involvement within these groups (Table 4). Regarding to the affected site, ileocecal lesions (the most common location of intestinal BD) were observed in all patients, and intestinal lesions in other parts of the intestine were also observed equally in both groups. There was no significant difference in the size, distribution pattern and shape between the two groups.

Regarding previous or current medication, all patients had received non-immunosuppressive treatment, such as colchicine or 5-aminosalicylic acid (5-ASA) (Table 5). Colchicine is highly effective in patients with FMF and *MEFV* gene-associated enterocolitis. Therefore, we assessed the effectiveness of colchicine in patients with intestinal BD. Colchicine was administered to most patients of both groups (92% and 75% in the *MEFV*+ and *MEFV*− groups, respectively). In the *MEFV*+ group, only two patients (17%) were controlled with colchicine and 5-ASA without the use of immunosuppressive drugs. In the *MEFV*− group, three patients (75%) showed significant response to colchicine with or without 5-ASA. In the *MEFV*+ group, eight patients (67%) required treatment with tumor necrosis factor-alpha (TNFα) inhibitors and three patients (25%) underwent intestinal resection due to hemorrhage or perforation of the intestine. Moreover, two of three patients required multiple surgeries due to intestinal perforation following the first operation. In the *MEFV*− group, only one patient (25%) required therapy with TNFα inhibitors and none (0%) underwent surgery. These clinical courses indicated that patients in the *MEFV*+ group were more refractory to treatment than those in the *MEFV*− group.

### 3.3. Cytokine Expression Pattern among Patients with Intestinal BD

**Figure 2:**
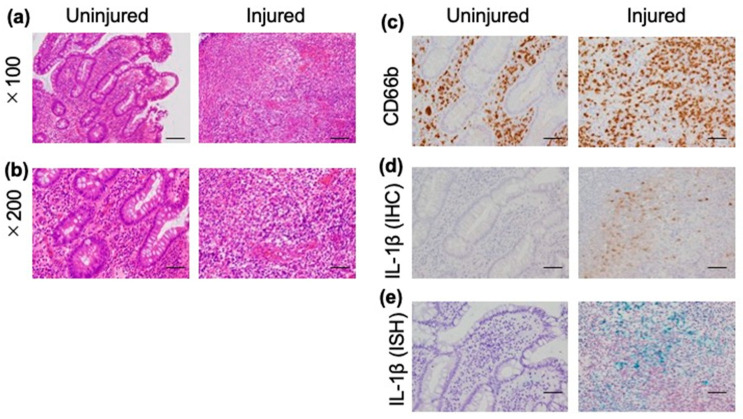
Expression of IL-1β in intestinal lesions of patients with Behçet’s disease. Histological findings of a representative case (No. 5 in Table 5). Hematoxylin and eosin (HE) staining showed inflammatory cell infiltration of both injured and uninjured areas (**a**,**b**). Immunohistochemistry (IHC) staining with an anti-CD66b antibody revealed infiltration of both injured and uninjured areas by neutrophils (**c**). IHC with an anti-IL-1β antibody (**d**) and in situ hybridization (ISH) with an IL-1β antibody (green) (**e**) revealed that IL-1β was only expressed in injured areas. Bar indicates 100 μm (**a**) and 50 μm (**b**–**e**). IL-1β, interleukin-1β.

The *MEFV* gene encodes pyrin, which regulates the overactivation of the inflammasome. This process leads to the controlled production of mature IL-1β and IL-18. To assess the involvement of IL-1β and IL-18 in intestinal lesions of BD, we performed IHC staining of biopsy samples from intestinal lesions using an anti-IL-1β antibody and an anti-IL-18 antibody in eight patients with intestinal BD (four patients with *MEFV* gene mutations, four patients without *MEFV* gene mutations) (Figure 2 and Supplementary Figure S1). We observed infiltration of both uninjured and injured areas by inflammatory cells (Figure 2a,b), particularly CD66b-positive neutrophils (Figure 2c). IHC with an anti-IL-1β antibody revealed that IL-1β was highly expressed only in injured areas (Figure 2d), while IL-18 was not expressed in either of the areas. Additionally, we performed in situ hybridization to further assess the expression of IL-1β. Similar to the findings obtained from the IHC study, IL-1β was highly expressed only in injured areas (Figure 2e), suggesting that IL-1β may be involved in the formation of ulcers in patients with *MEFV*+ intestinal BD (Figure 2d,e and Supplementary Figure S1a).

## 4. Discussion

The results of this study revealed a high incidence of *MEFV* gene mutations in patients with intestinal BD (75%). The incidence was relatively higher than those of BD patients without intestinal lesions (50%) and healthy controls (38%). In addition, we compared the clinical features of intestinal BD with or without these mutations. Most *MEFV*+ patients were relatively refractory and required immunosuppressive therapy or operation. Interestingly, in situ hybridization showed a high expression of IL-1β only in injured areas, suggesting that IL-1β may be involved in the formation of ulcers in patients with *MEFV*+ intestinal BD.

Genome-wide association studies have demonstrated that genetic factors are associated with the onset of BD [10,20,21]. Although there may be an association between subtypes of BD and genetic factors, the relationship between intestinal lesions and genetic factors remains unclear and has been investigated by a limited number of studies [4,7].

BD and FMF share several clinical features. Both diseases are characterized by recurrent episodes and respond well to treatment with colchicine. The Mediterranean area is a hotspot for these conditions, and the coexistence of these diseases has been reported in some patients [8,9]. Furthermore, the gene responsible for FMF (*MEFV*) was identified as a susceptibility gene for BD by Kirino et al. [10].

FMF is considered an autosomal-recessive inherited disease caused by mutations in the *MEFV* gene. FMF is primarily involved in inflammation of the serous membrane. Therefore, previously, it was unclear whether FMF can cause inflammation within the intestinal mucosa. However, cases of inflammatory bowel disease associated with *MEFV* gene mutations have been reported and have attracted attention as a new disease concept, particularly in Japan, owing to the significant therapeutic effect of colchicine. Therefore, *MEFV* gene mutations may be associated with inflammation in the intestinal mucosa. Recently, Ishikawa et al. reported that mutations in the *MEFV* gene may be a disease modifier in neuro-BD or neuro-Sweet disease [13]. Nevertheless, the role of *MEFV* gene mutations in intestinal BD has not been investigated yet. In the present study, we showed that the rate of *MEFV* gene mutations in patients with intestinal BD was significantly higher than that of healthy controls. Therefore, the mutated *MEFV* gene may be a susceptibility gene for intestinal lesions and modify the disease subtypes of BD. Although the difference was not statistically significant due to the small sample size, the rate of mutations in patients with intestinal BD was relatively higher than that noted in BD patients without intestinal lesions. Careful follow-up of BD patients without intestinal lesions who carry these gene mutations is required because of the risk of developing such lesions in the future.

We also analyzed the association between the clinical course and these gene mutations. Based on the good response of FMF and *MEFV* gene-related enterocolitis to treatment with colchicine, we predicted that patients with intestinal BD harboring *MEFV* gene mutations also respond well to this agent. Contrary to our hypothesis, most patients with intestinal BD carrying *MEFV* gene mutations required immunosuppressive therapy. All three cases, which were refractory to medical therapy and required intestinal resection due to hemorrhage or intestinal perforation, harbored *MEFV* gene mutations. These clinical courses indicate that mutations in the *MEFV* gene may be a factor involved in the development of resistance to treatment.

IL-1β is the principal proinflammatory cytokine, which induces the expression of numerous chemokines and activates both innate and adaptive immune responses [22]. It is mainly elicited from myeloid cells, such as macrophages, monocytes, and neutrophils. The activation of neutrophils is one of the major etiologies of BD, and IL-1β plays a critical role in this disease [23,24]. Indeed, recent several case reports and some studies showed that IL-1β inhibitors were effective against treatment-refractory BD [25,26]. However, thus far, the potential involvement of IL-1β in intestinal lesions of BD has not been examined. In this study, we assessed the expression of IL-1β in intestinal lesions through IHC and in situ hybridization experiments. The findings showed that IL-1β was only expressed in injured areas, indicating that it may play an essential role in the formation of ulceration in intestinal BD. Recently, a study showed that the high expression of IL-1β in the intestinal mucosa was associated with resistance to corticosteroid therapy and treatment with TNFα inhibitors in inflammatory bowel diseases [27,28]. Therefore, the overexpression of IL-1β in the intestinal mucosa could also contribute to treatment refractoriness in intestinal BD. Owing to the small number of samples, we did not observe a significant difference in the expression of IL-1β between patients with and without *MEFV* gene mutations. Nevertheless, mutations in the *MEFV* gene may contribute to the expression of IL-1β in intestinal lesions, leading to a treatment-refractory clinical course in these patients.

Colchicine is effective against *MEFV* gene mutation-associated diseases (e.g., FMF) and cases of *MEFV* gene-related enterocolitis. However, in our cohort, monotherapy with colchicine was not effective in most patients with intestinal BD harboring mutations in the *MEFV* gene. The overactivation of innate immune responses leading to the overexpression of IL-1β is the primary pathogenic factor for BD. Therefore, colchicine is effective in most patients with BD and used for the initial treatment of this disease [29,30]. However, in the presence of mutations in the *MEFV* gene and excessive IL-1β, the disease may be uncontrollable with colchicine alone. Thus, more aggressive treatment, including biologics or surgery, may be required. The use of anti-IL-1β inhibitors may be an option for such patients who are refractory to treatment.

This study had some limitations. Firstly, this was a retrospective study conducted in a single center. Therefore, the sample size was relatively small due to disease rarity, and there was referral filter bias. Further multicenter studies with adjustment by clinical confounding factors are warranted to confirm the present findings. Secondly, ileocolonoscopy was not performed in all patients with BD. Therefore, some BD patients without intestinal lesions may have ulceration in the intestine. However, there were no complaints of abdominal symptoms by the patients without intestinal lesions. Despite these limitations, the prevalence of mutations in the *MEFV* gene was high in patients with intestinal BD, and the treatment of these patients was difficult. Therefore, combination therapies were required for patients with intestinal BD harboring *MEFV* gene mutations, and IL-1β inhibitors may be a treatment option for patients with refractory intestinal BD.

## 5. Conclusions

In this study, we showed a high prevalence of *MEFV* gene mutations in patients with intestinal BD. Therefore, we recommend patients with BD harboring *MEFV* gene mutations to perform the endoscopic examination to rule out the intestinal lesions. In addition, because *MEFV* gene mutation-positive patients with intestinal BD showed refractory clinical courses, multidisciplinary treatment, including biologics, should be considered for such patients.

## Figures and Tables

**Table 1:** Background of patients with Behçet’s disease (BD) (*n* = 26).

Characteristic	*n*
Age at diagnosis, years, median (range)	34 (17–80)
BD duration, years, median (range)	8.5 (1–20)
Sex	
Male	19 (73%)
Female	7 (27%)
Clinical subtype of BD	
Complete type	5 (19%)
Incomplete type	16 (62%)
Suspected	5 (19%)
Clinical manifestations	
*Major symptoms*	
Oral ulcers	26 (100%)
Skin lesions	17 (65%)
Eye lesions	13 (50%)
Genital ulcers	10 (38%)
*Minor symptoms*	
Intestinal lesions	16 (62%)
Arthritis/arthralgia	15 (57%)
Vascular lesions	5 (19%)
Central nervous system lesions	5 (19%)
Epididymitis	2 (8%)
Human leukocyte antigen (HLA)	
B51	10 (38%)
A26	6 (23%)
B51 and A26	2 (8%)

**Table 2:** Frequency of *MEFV* mutations and genotypes of patient with Behçet’s disease (BD).

Mutation	Genotype	Intestinal BD (*n* = 16)	BD without Intestinal Lesions (*n* = 10)
Homozygous	L110P/E148Q	0 (0%)	2 (20%)
	E148Q	3 (19%)	2 (20%)
Compound Heterozygous	E148Q/P369S/R408Q	1 (6%)	0 (0%)
	P369S/R408Q	1 (6%)	0 (0%)
Heterozygous	E148Q	6 (38%)	0 (0%)
	G304S	1 (6%)	1 (10%)
No mutations		4 (25%)	5 (50%)

*MEFV*, Mediterranean fever.

**Table 3:** Comparison of characteristics between with and without MEFV gene mutation in patients with intestinal Behçet’s disease.

*MEFV* Gene Mutation	*MEFV*− (*n* = 4)	*MEFV*+ (*n* = 12)	*p*-Value
Age, years, median [range]	47.5 [21–72]	41 [21–80]	1.000
Clinical manifestations, *n* (%)			
*Major symptoms*			
Oral ulcers	4 (100%)	12 (100%)	-
Skin lesions	4 (100%)	4 (33%)	0.077
Eye lesions	2 (50%)	2 (17%)	0.547
Genital ulcers	1 (25%)	3 (25%)	1.000
*Minor symptoms*			
Arthritis/arthralgia	3 (75%)	7 (58%)	1.000
Vascular lesions	2 (50%)	2 (17%)	0.547
Central nervous system lesions	0 (0%)	4 (33%)	0.529
Epididymitis	0 (0%)	2 (17%)	1.000
HLA, *n* (%)			
B51	2 (50%)	5 (42%)	1.000
A26	2 (50%)	4 (33%)	0.245

*MEFV*, Mediterranean fever; HLA, human leukocyte antigen.

**Table 4:** Comparison of endoscopic findings between with and without *MEFV* gene mutation in patients with intestinal Behçet’s disease.

*MEFV* Gene Mutation	*MEFV*− (*n* = 4)	*MEFV*+ (*n* = 12)	*p*-Value
Location, *n* (%)			
Esophagus	2 (50%)	5 (42%)	1.000
Small intestine (excluding terminal ileum)	0 (0%)	1 (8%)	1.000
Ileocecum	4 (100%)	12 (100%)	
Colon	1 (25%)	0 (0%)	0.250
Size (cm), *n* (%)			0.180
≦1	1 (25%)	2 (17%)	
>1, ≦3	2 (50%)	5 (42%)	
>3	1 (25%)	5 (42%)	
Distribution pattern, *n* (%)			1.000
Single	1 (25%)	3 (25%)	
Multiple	3 (75%)	9 (75%)	
Shape, *n* (%)			0.180
Round/oval	2 (50%)	6 (50%)	
Geographic	1 (25%)	0 (0%)	
Volcano	1 (25%9	6 (0%)	

*MEFV*, Mediterranean fever.

**Table 5:** *MEFV* gene mutation of patients with intestinal Behçet’s disease and treatments.

		*MEFV* Gene Mutation					Treatment								
		Exon 2		Exon 3									Biologics		
	No.	L110P	E148Q	G304S	P369S	R408Q	5-ASA	Col	PSL	AZA	MTX	CyA	IFX	ADA	Op
*MEFV*+ group	1						◯	◯							
	2		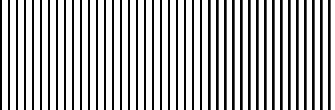				◯	◯							
	3							◯	◯						
	4						◯		◯						
	5						◯	◯	◯	◯			◯		
	6						◯	◯	◯	◯				◯	
	7						◯	◯	◯	◯			◯		
	8		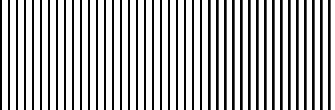				◯	◯	◯					◯	
	9		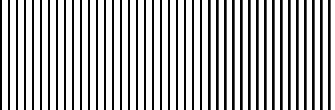					◯			◯		◯		
	10						◯	◯	◯	◯			◯	◯	◯
	11						◯	◯	◯				◯		◯
	12						◯	◯	◯				◯	◯	◯
*MEFV*− group	13						◯								
	14						◯	◯							
	15						◯	◯							
	16							◯			◯	◯	◯		

Stripe squares indicate positive for homozygous mutation in *MEFV* gene. Gray squares indicate positive for heterozygous mutation in *MEFV* gene. *MEFV*, Mediterranean fever gene; 5-ASA, 5-aminosalicylic acid; Col, colchicine; PSL, prednisolone; AZA, azathioprine; MTX, methotrexate; CyA, cyclosporine; IFX, infliximab; ADA, adalimumab; Op, operation. Circles in treatment category indicate previous or current treatment.

## Data Availability

All data underlying this study are included in this article. Further inquiries can be directed to the corresponding author.

## Supplementary Materials

The following supporting information can be downloaded at: https://www.mdpi.com/article/10.3390/jcm12093131/s1, Figure S1: Expression of interleukin-1β (IL-1β) and IL-18 in biopsy samples.
