# Supplementary material for: Possible Association of Mutations in the MEFV Gene with the Intestinal Phenotype of Behçet’s Disease and Refractoriness to Treatment
Source: J Clin Med. 2023 Apr 26;12(9):3131. doi: 10.3390/jcm12093131 (PMC10179346; doi:10.3390/jcm12093131)
Supplement: Supplementary file 1 [file jcm-12-03131-s001.zip › jcm-2285455-supplementary.pptx]

## Slide 1
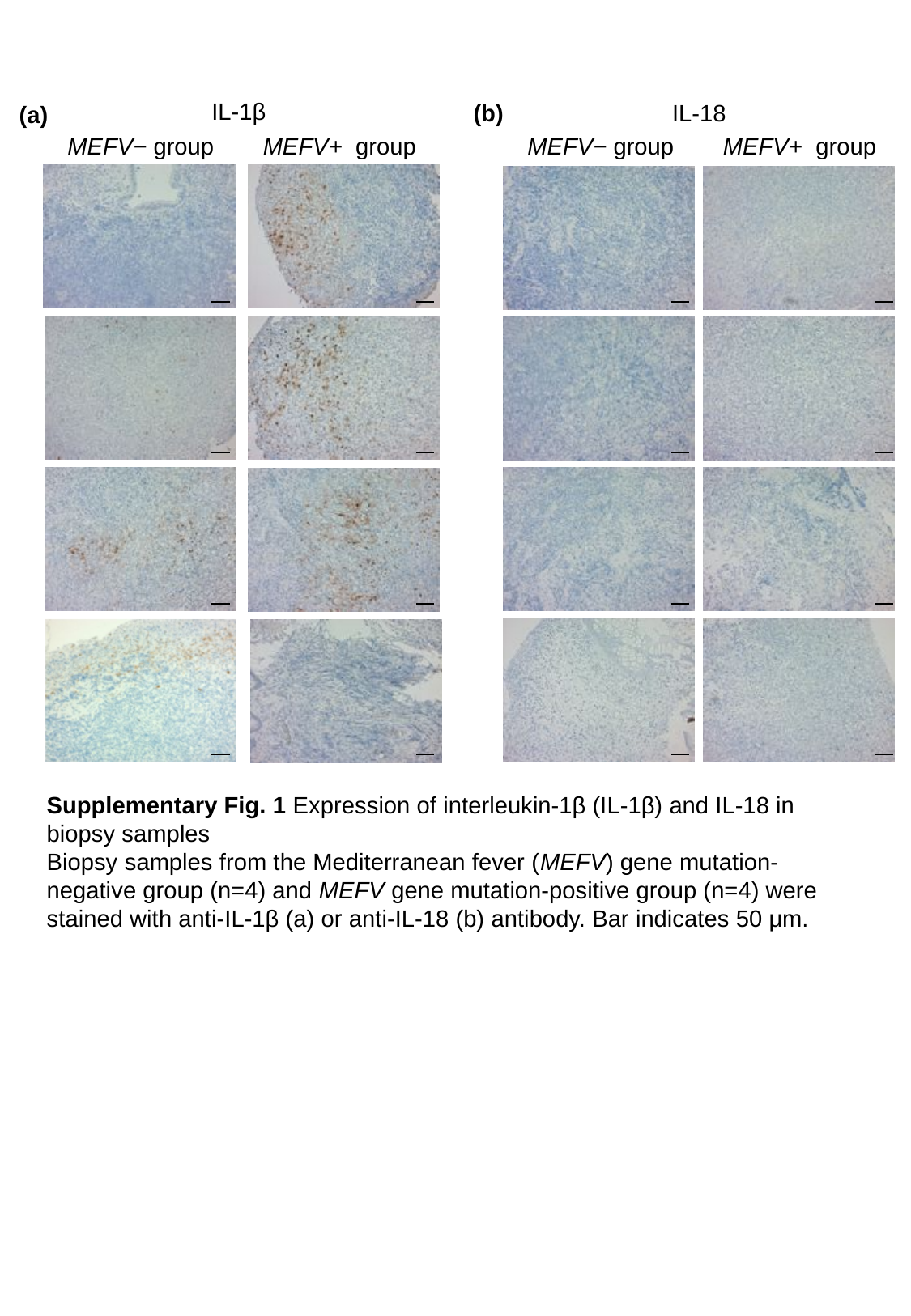

IL-1β
(b)
IL-18
(a)
MEFV− group
MEFV+ group
MEFV− group
MEFV+ group
Supplementary Fig. 1 Expression of interleukin-1β (IL-1β) and IL-18 in biopsy samples
Biopsy samples from the Mediterranean fever (MEFV) gene mutation-negative group (n=4) and MEFV gene mutation-positive group (n=4) were stained with anti-IL-1β (a) or anti-IL-18 (b) antibody. Bar indicates 50 μm.
